# Supplementary material for: Evaluation of Blood Intercellular Adhesion Molecule-1 (ICAM-1) Level in Obstructive Sleep Apnea: A Systematic Review and Meta-Analysis
Source: Medicina (Kaunas). 2022 Oct 21;58(10):1499. doi: 10.3390/medicina58101499 (PMC9607021; doi:10.3390/medicina58101499)
Supplement: Supplementary file 1 [file medicina-58-01499-s001.zip › medicina-1889057-supplementary.pdf]

Table S1: Search strategy in PubMed

No.	Search items
#1	(“obstructive sleep apnea-hypopnea syndrome” [Title/Abstract] OR “OSAHS” [Title/Abstract] OR “obstructive Sleep Apnea”[Title/Abstract] OR “sleep apnea”[Title/Abstract] OR “OSA”[Title/Abstract] OR “obstructive sleep apnea syndrome” [Title/Abstract] OR “OSAS” [Title/Abstract])
#2	(“Sleep Apnea, Obstructive” [MeSH Terms] OR “Apnea”[MeSH Terms])
#3	#1OR #2
#4	(“inter-cellular adhesion molecule*”[Title/Abstract] OR “intercellular adhesion molecule*”[Title/Abstract] OR “ICAM” [Title/Abstract] OR “cell adhesion molecule*”[Title/Abstract] OR “CAM” [Title/Abstract])
#5	(“Cell Adhesion Molecules”[MeSH Terms])
#6	#4 OR #5
#7	#3 AND #6

Table S2: Quality score of the studies based on three domains (Selection, Comparability, and Exposure)

Study, publication year	Selection (Max 4 point)				Comparability (Max 2 point)		Exposure (Max 3 point)			Total point
	1A	2A	3A	4A	1B	2B	1C	2C	3C	
El-Solh, 2002 [43]	*	*	*	*	*	*	*	*	*	9
Ohga, 2003 [44]	*	*	*	*	*	*	*	*	*	9
Bravo, 2007 [45]	*	*	*	*	*	*	*	*	*	9
Ursavaş, 2007 [46]	*	*	*	-	*	*	*	*	*	8
Carpagnano, 2010 [47]	*	*	*	-	*	-	*	*	*	7
Zamarrón, 2011 [48]	*	*	*	*	*	-	*	*	*	8
Zhi, 2011 [49]	*	*	*	*	*	-	*	*	*	8
Jurado-Gamez, 2012 [50]	*	*	*	*	*	*	*	*	-	8
Chen, 2015 [51]	*	*	*	*	*	*	*	*	*	9
da Silva Araújo, 2015 [31]	*	*	*	-	-	-	*	*	*	6
Chang, 2017 [52]	*	*	*	*	*	*	*	*	*	9
Jin, 2017 [53]	*	*	*	*	*	*	*	*	*	9
Xiao, 2017 [30]	*	*	*	-	*	*	*	*	*	8
Santamaria-Martos, 2018 [29]	*	*	*	-	-	-	*	*	*	6
Sun, 2019 [54]	*	*	*	-	*	*	*	*	*	8
Nikitidou, 2021 [55]	*	*	*	-	*	*	*	*	*	8
Sun, 2022 [56]	*	*	-	-	*	-	*	*	*	6

Each asterisk donates one point. 1A: Adiquate definition of case. 2A: Representitavness of cases. 3A: Selection of control. 4A: Definition of control. 1B: Control for age. 2B: Control for sex. 1C: Exposure assessment. 2C: Same method of ascertainment for cases and controls. 3C: Nonrsponse rate.

Study or Subgroup	Case Mean	Case SD	Case Total	Control Mean	Control SD	Control Total	Weight	Std. Mean Difference IV, Random, 95% CI
Bravo, 2007	263	46.9	22	221	38.01	20	4.9%	0.96 [0.32, 1.60]
Carpagnano, 2010	100.1	3.6	12	93.3	2.6	10	4.5%	2.05 [0.98, 3.12]
Chang, 2017	214.6	78.1	121	183.9	33	27	5.1%	0.42 [0.00, 0.84]
Chen, 2015	206.93	81.03	20	176.67	35.24	14	4.9%	0.45 [-0.25, 1.14]
da Silva Araújo, 2015	105.23	31.19	33	101.38	33.23	20	5.0%	0.12 [-0.44, 0.67]
El-Solh, 2002	367.4	85.2	15	252.8	68.4	15	4.8%	1.44 [0.63, 2.26]
Jin, 2017	357.92	10.52	100	91.68	53.29	50	4.5%	8.32 [7.31, 9.33]
Nikitidou, 2021	471.2	204.5	20	243.6	39.9	10	4.7%	1.30 [0.47, 2.14]
Ohga, 2003	448.57	153.79	20	222.14	114.79	10	4.7%	1.55 [0.68, 2.41]
Santamaria-Martos, 2018 (mild)	148.37	77.8	109	90.55	66.32	132	5.2%	0.80 [0.54, 1.07]
Santamaria-Martos, 2018 (mod/sev)	88	75.68	119	90.55	66.32	132	5.2%	-0.04 [-0.28, 0.21]
Sun, 2019	570.17	366.45	44	147.39	185.94	24	5.0%	1.32 [0.78, 1.87]
Sun, 2022 (mild)	575.6	388.09	29	149.21	255.45	56	5.0%	1.38 [0.88, 1.87]
Sun, 2022 (mod)	496.02	331.82	33	149.21	255.45	56	5.1%	1.20 [0.74, 1.67]
Sun, 2022 (sev)	624.6	357.45	99	149.21	255.45	56	5.1%	1.46 [1.09, 1.82]
Ursavaş, 2007	480.1	216.7	39	303.4	98.6	34	5.1%	1.02 [0.53, 1.51]
Xiao, 2017 (mild)	361.7	21.84	31	342.71	17.76	31	5.0%	0.94 [0.42, 1.47]
Xiao, 2017 (mod)	518.41	30.46	31	342.71	17.76	31	4.1%	6.96 [5.60, 8.32]
Xiao, 2017 (sev)	711.27	32.67	31	342.71	17.76	31	2.6%	13.84 [11.27, 16.41]
Zamarrón, 2011	251.67	69.62	20	221	48.15	18	4.9%	0.50 [-0.15, 1.14]
Zhi, 2011	118.3	18.3	20	55.3	19	20	4.6%	3.31 [2.33, 4.29]
**Total (95% CI)**			**968**			**797**	**100.0%**	**2.00 [1.41, 2.59]**

Heterogeneity: Tau² = 1.70; Chi² = 499.18, df = 20 (P < 0.00001); I² = 96%
Test for overall effect: Z = 6.69 (P < 0.00001)

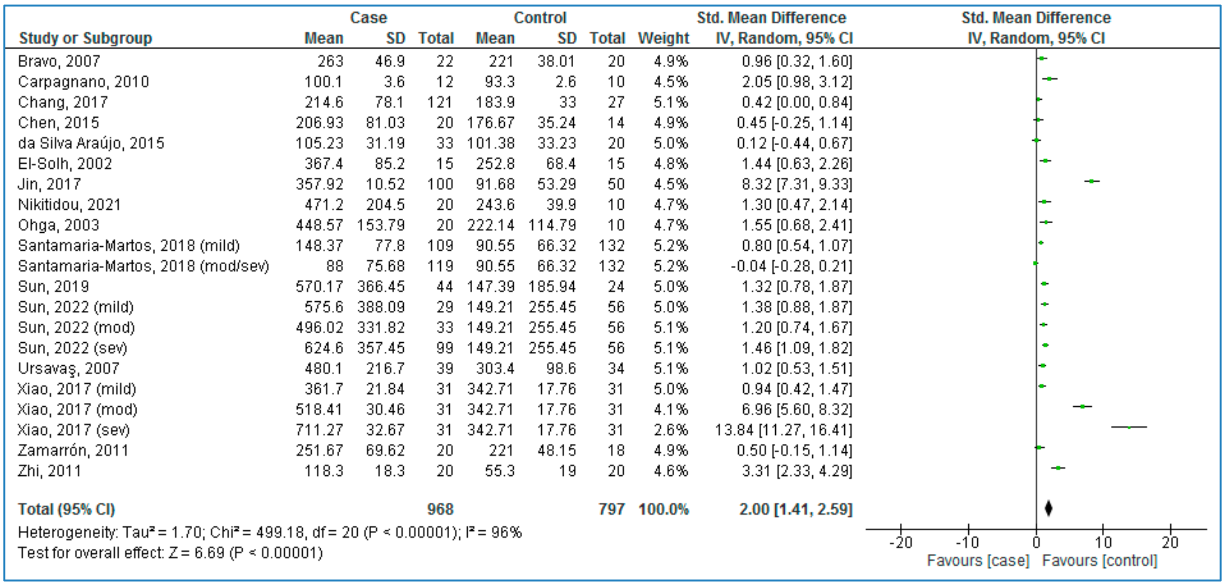

Figure S1: Random-effects forest plot analysis. Serum/plasma intercellular adhesion molecule-1 levels in adults with obstructive sleep apnea compared to controls.

	Severe			Mild/Moderate				Std. Mean Difference
Study or Subgroup	Mean	SD	Total	Mean	SD	Total	Weight	IV, Random, 95% CI
Jurado-Gamez, 2012	435	148.89	37	257.33	77.78	31	21.2%	1.44 [0.90, 1.98]
Sun, 2022 (mild)	624.6	357.45	99	575.6	388.09	29	21.3%	0.13 [-0.28, 0.55]
Sun, 2022 (mod)	624.6	357.45	99	496.02	331.82	33	21.4%	0.36 [-0.03, 0.76]
Xiao, 2017 (mild)	711.27	32.67	31	361.7	21.84	31	16.3%	12.42 [10.11, 14.74]
Xiao, 2017 (mod)	711.27	32.67	31	518.41	30.64	31	19.8%	6.01 [4.81, 7.21]
**Total (95% CI)**			**297**			**155**	**100.0%**	**3.62 [1.74, 5.51]**

Heterogeneity: Tau² = 4.27; Chi² = 188.87, df = 4 (P < 0.00001); I² = 98%
Test for overall effect: Z = 3.78 (P = 0.0002)

Std. Mean Difference
IV, Random, 95% CI

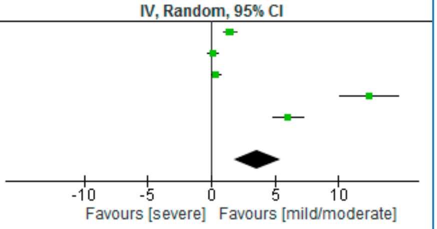

Figure S2: Random-effects forest plot analysis. Serum/plasma intercellular adhesion molecule-1 levels in adults with severe compared to mild/moderate obstructive sleep apnea.

Study name	Statistics with study removed						
	Point	Standard error	Variance	Lower limit	Upper limit	Z-Value	p-Value
Bravo, 2007	2.11	0.32	0.10	1.49	2.73	6.645	0.000
Carpagnano, 2010	2.04	0.31	0.10	1.43	2.65	6.547	0.000
Chang, 2017	2.15	0.32	0.10	1.51	2.78	6.658	0.000
Chen, 2015	2.13	0.32	0.10	1.51	2.75	6.752	0.000
da Silva Araújo, 2015	2.15	0.32	0.10	1.53	2.77	6.804	0.000
El-Solh, 2002	2.08	0.31	0.10	1.46	2.69	6.604	0.000
Jin, 2017	1.63	0.24	0.06	1.15	2.11	6.675	0.000
Nikitidou, 2021	2.08	0.31	0.10	1.47	2.70	6.626	0.000
Ohga, 2003	2.07	0.31	0.10	1.46	2.69	6.596	0.000
Santamaria-Martos, 2018 (mild)	2.15	0.34	0.12	1.48	2.82	6.304	0.000
Santamaria-Martos, 2018 (mod/sev)	2.17	0.32	0.10	1.54	2.79	6.797	0.000
Sun, 2019	2.09	0.32	0.10	1.47	2.72	6.562	0.000
Sun, 2022 (mild)	2.09	0.32	0.10	1.46	2.72	6.534	0.000
Sun, 2022 (mod)	2.10	0.32	0.10	1.47	2.73	6.536	0.000
Sun, 2022 (sev)	2.10	0.33	0.11	1.46	2.73	6.431	0.000
Ursava?, 2007	2.11	0.32	0.10	1.48	2.74	6.577	0.000
Xiao, 2017 (mild)	2.11	0.32	0.10	1.49	2.74	6.608	0.000
Xiao, 2017 (mod)	1.80	0.29	0.08	1.23	2.36	6.248	0.000
Xiao, 2017 (sev)	1.69	0.28	0.08	1.15	2.24	6.109	0.000
Zamarrón, 2011	2.13	0.32	0.10	1.51	2.75	6.733	0.000
Zhi, 2011	1.97	0.31	0.09	1.37	2.57	6.437	0.000
	2.04	0.30	0.09	1.45	2.64	6.727	0.000

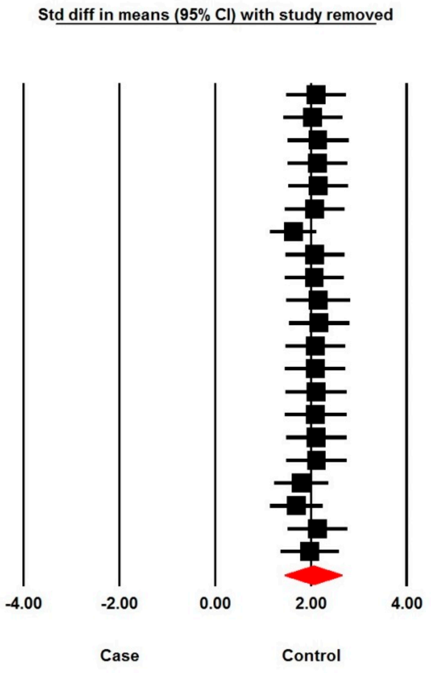

Figure S3: One-study-removed analysis of serum/plasma intercellular adhesion molecule-1 levels in adults with obstructive sleep apnea compared to controls.

Study name	Cumulative statistics						
	Point	Standard error	Variance	Lower limit	Upper limit	Z-Value	p-Value
Bravo, 2007	0.98	0.33	0.11	0.34	1.62	2.994	0.003
Carpagnano, 2010	1.48	0.57	0.33	0.36	2.60	2.586	0.010
Chang, 2017	1.06	0.43	0.18	0.23	1.89	2.491	0.013
Chen, 2015	0.88	0.31	0.09	0.27	1.48	2.854	0.004
da Silva Araújo, 2015	0.70	0.26	0.07	0.19	1.21	2.701	0.007
El-Solh, 2002	0.83	0.25	0.06	0.33	1.33	3.246	0.001
Jin, 2017	1.96	0.80	0.64	0.39	3.53	2.447	0.014
Nikitidou, 2021	1.88	0.71	0.50	0.49	3.27	2.651	0.008
Ohga, 2003	1.84	0.64	0.41	0.59	3.10	2.885	0.004
Santamaria-Martos, 2018 (mild)	1.72	0.49	0.24	0.76	2.68	3.512	0.000
Santamaria-Martos, 2018 (mod/sev)	1.54	0.42	0.17	0.72	2.36	3.693	0.000
Sun, 2019	1.52	0.38	0.15	0.76	2.27	3.944	0.000
Sun, 2022 (mild)	1.50	0.36	0.13	0.81	2.20	4.223	0.000
Sun, 2022 (mod)	1.48	0.33	0.11	0.83	2.12	4.496	0.000
Sun, 2022 (sev)	1.47	0.30	0.09	0.88	2.06	4.859	0.000
Ursava?, 2007	1.44	0.28	0.08	0.88	1.99	5.080	0.000
Xiao, 2017 (mild)	1.40	0.27	0.07	0.88	1.93	5.267	0.000
Xiao, 2017 (mod)	1.67	0.29	0.08	1.10	2.24	5.760	0.000
Xiao, 2017 (sev)	2.06	0.32	0.10	1.43	2.69	6.443	0.000
Zamarrón, 2011	1.97	0.31	0.09	1.37	2.57	6.437	0.000
Zhi, 2011	2.04	0.30	0.09	1.45	2.64	6.727	0.000
	2.04	0.30	0.09	1.45	2.64	6.727	0.000

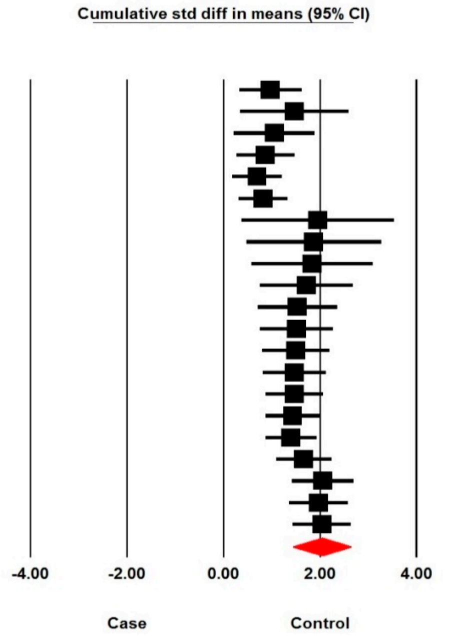

Figure S4: Cumulative analysis of serum/plasma intercellular adhesion molecule-1 levels in adults with obstructive sleep apnea compared to controls.

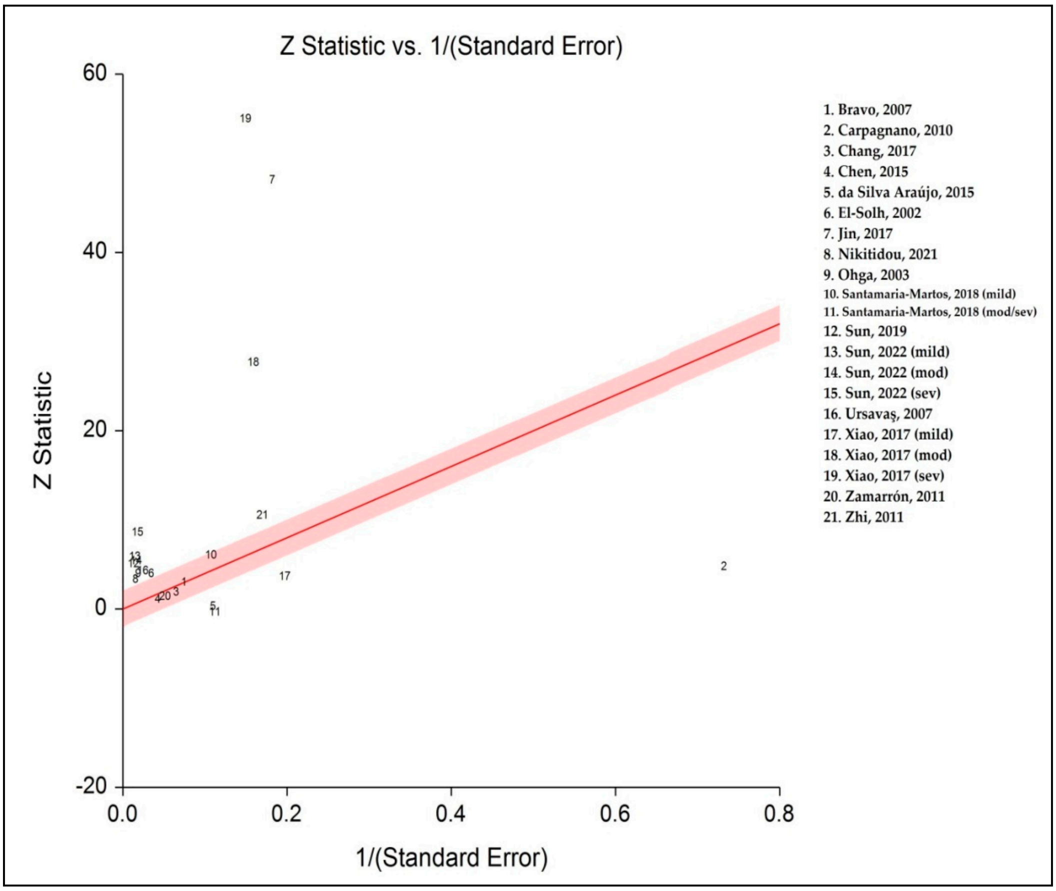

Figure S5: Galbraith (or Radial plot) of serum/plasma intercellular adhesion molecule-1 levels in adults with obstructive sleep apnea compared to controls. Studies with the largest weight are closest to the Y axis. Studies within the limits (95% confidence interval) are interpreted as homogeneous. Studies outside the limits (95% confidence interval) may be outliers.

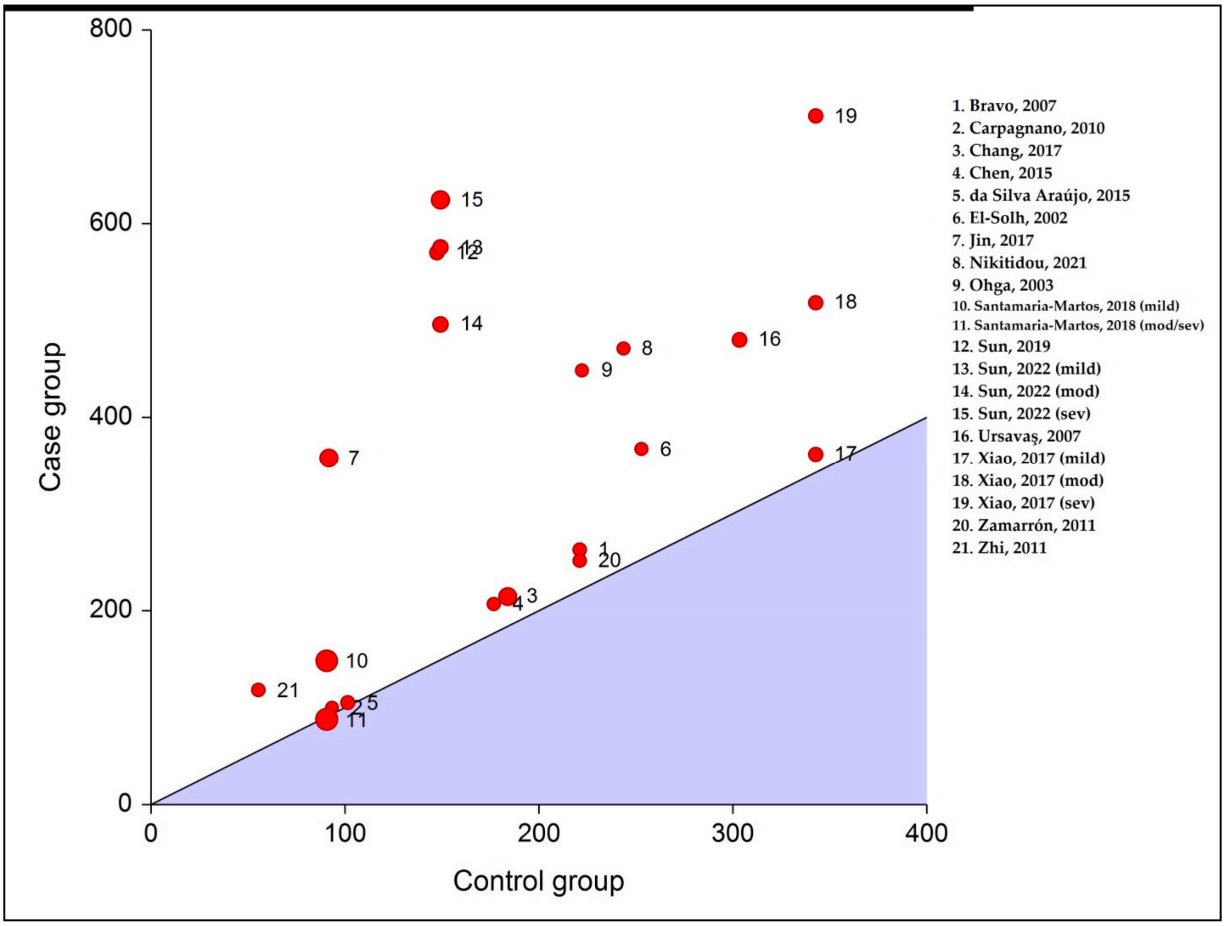

Figure S6: L'Abbe plot of serum/plasma intercellular adhesion molecule-1 levels in adults with obstructive sleep apnea compared to controls. Homogenous studies will be arranged along the diagonal line.

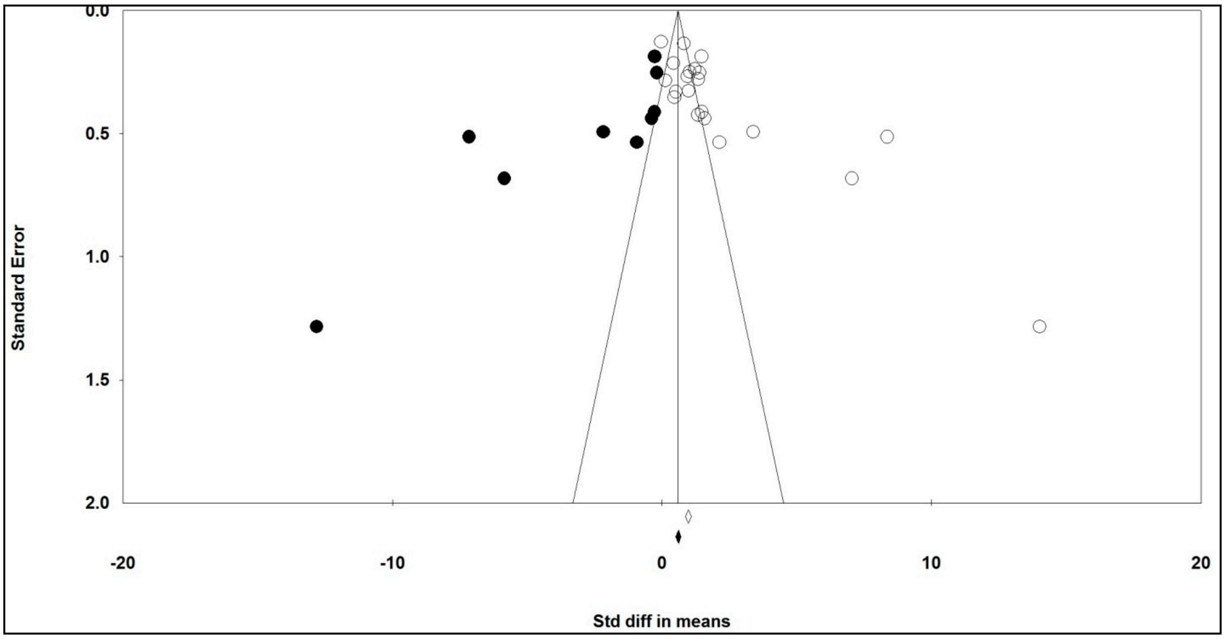

Figure S7: Funnel plot of serum/plasma intercellular adhesion molecule-1 levels in adults with obstructive sleep apnea compared to controls.

Table S3: The results of trim-and-fill method.

Sample	Value	Studies trimmed	Fixed-effects			Random-effects			Q value
			Point estimate	Lower limit	Upper limit	Point estimate	Lower limit	Upper limit	
Serum/plasma	Observed	-	0.967	0.856	1.077	2.043	1.448	2.639	520.220
	Adjusted	9	0.598	0.498	0.698	0.665	0.029	1.301	1071.807
